# EEG functional connectivity analysis in the source space

**DOI:** 10.1016/j.dcn.2022.101119

**Published:** 2022-06-09

**Authors:** Wanze Xie, Russell T. Toll, Charles A. Nelson

**Affiliations:** a School of Psychological and Cognitive Sciences, Peking University, China; b PKU-IDG/McGovern Institute for Brain Research, Peking University, China; c Beijing Key Laboratory of Behavior and Mental Health, Peking University, China; d Department of Psychiatry, University of Texas Southwestern Medical Centre at Dallas, USA; e Boston Children’s Hospital, Boston, MA, USA; f Harvard Medical School, Boston, MA, USA; g Harvard Graduate School of Education, Cambridge, MA, USA

**Keywords:** EEG, Source analysis, Source-space functional connectivity

## Abstract

There is a growing interest in using electroencephalography (EEG) and source modeling to investigate functional interactions among cortical processes, particularly when dealing with pediatric populations. This paper introduces two pipelines that have been recently used to conduct EEG FC analysis in the cortical source space. The analytic streams of these pipelines can be summarized into the following steps: 1) cortical source reconstruction of high-density EEG data using realistic magnetic resonance imaging (MRI) models created with age-appropriate MRI templates; 2) segmentation of reconstructed source activities into brain regions of interest; and 3) estimation of FC in age-related frequency bands using robust EEG FC measures, such as weighted phase lag index and orthogonalized power envelope correlation. In this paper we demonstrate the two pipelines with resting-state EEG data collected from children at 12 and 36 months of age. We also discuss the advantages and limitations of the methods/techniques integrated into the pipelines. Given there is a need in the research community for open-access analytic toolkits that can be used for pediatric EEG data, programs and codes used for the current analysis are made available to the public.

## 1 Introduction

Progress made in developing novel neuroimaging tools offers up the opportunity to investigate the dynamic interregional communications in the brain and their development over childhood. Studies with functional magnetic resonance imaging (fMRI) have advanced our understanding of the development of functional brain networks (Gilmore et al., 2018). However, there are still challenges to collect fMRI data from awake young children (e.g., infants), including head and body motion, fussiness of the participant, and high acoustic noise level. Furthermore, hemodynamic signals provide an indirect measure of neural activity. In contrast, electroencephalography (EEG) offers a comparatively inexpensive and easy-to-use alternative to study brain development in pediatric populations (Xie and Nelson, 2021). More importantly, EEG reflects a direct measure of neural activity with high temporal resolution, which allows for assessing neural oscillations within specific frequency bands that could reflect biophysical properties of local and large-scale network interactions. When combined with source modeling, EEG can be used to study functional interactions among cortical processes non-invasively (Michel and Murray, 2012). The advantages stemming from the nature of EEG recording make it a more practical tool to estimate functional connectivity (FC) while young children are performing cognitive tasks or in resting-state while they are awake.

The EEG FC is typically estimated by analyzing the correlation or coherence between dynamic signals recorded at multiple channels/electrodes on the scalp (Boersma et al., 2011, Miskovic et al., 2015). While previous findings of EEG FC at the scalp-level have provided insights into the development of brain networks, limited information about the underlying connections between brain regions can be obtained from scalp-level FC due to the issue of *volume conduction*, which is also referred to as *field spread*. Specifically, volume conduction refers to the transmission of electric fields from a cortical source (or dipole) through biological tissues towards measurement electrodes. Consequently, the electric fields generated by one dipole can be “visible” at multiple electrodes, i.e., field smearing on the scalp, due to the complexity of brain and head tissues through which the fields are transmitted. This will cause spurious correlation and synchronization values between electrodes. Therefore, the interpretation of the scalp-level connectivity requires considerable caution because the FC between two electrodes might reflect the activation generated by one brain region rather than two functionally connected regions. The effects of volume conduction on scalp-level EEG FC can be reduced by techniques like partial coherence field (Pascual-Marqui et al., 2011, Pascual-Marqui et al., 2011) and surface Laplacian (Carvalhaes and De Barros, 2015). An alternative way to ameliorate the impact is by analyzing the FC with reconstructed cortical source activities (Schoffelen and Gross, 2009), and meanwhile this method provides insights into the connections between cortical regions. To this end, people have leveraged the combination of cortical source reconstruction, FC estimation methods, age-appropriate MRI atlases, and high-density EEG recordings as a valid alternative to study the development of FC in various brain networks in young children (e.g., Bathelt et al., 2013; Tokariev et al., 2019, Tokariev et al., 2019).

Neuronal interactions in large-scale networks are represented by different forms of correlational metrics that reflect distinct facets of the interactions (Palva and Palva, 2012). Two forms of interareal correlations, phase-to-phase synchrony (PPS) and amplitude-to-amplitude correlation (AAC), which represent neuronal activity as phase and amplitude dynamics respectively, have been widely adopted in recent studies on source space FC. The PPS reflects how brain signals in two or more sources oscillate with a consistent sequence of relative phase angles, which regulate neuronal spiking and modulate (suppress or facilitate) interareal communication (Palva and Palva, 2011). The AAC, on the other hand, reflects characteristics in multi-second time scales that indicate co-modulations of overall neuronal activity (i.e., amplitude) levels and correlations in gross cortical excitability fluctuations (Hipp et al., 2012). The measures of PPS and AAC have been widely used with resting-state EEG data, but it is also possible to compute these measures at sub-second resolution for event-related (i.e., task-based) oscillations, especially those at higher frequencies (Karamzadeh et al., 2013, Miskovic and Keil, 2015). While phase-amplitude coupling is another metric to study EEG FC, it is beyond the scope of the current study and has been discussed elsewhere (Aru et al., 2015).

**Fig. 1:**
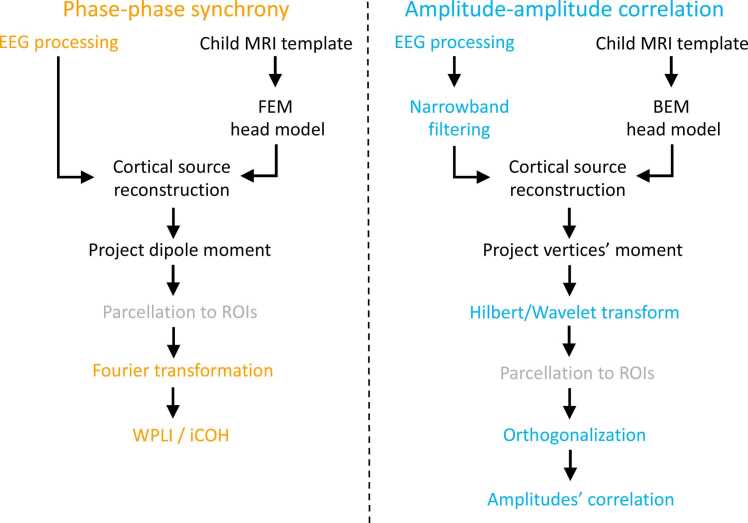
The processing stream of the pl_pps (left) and pl_aac (right) pipelines.

**Fig. 2:**
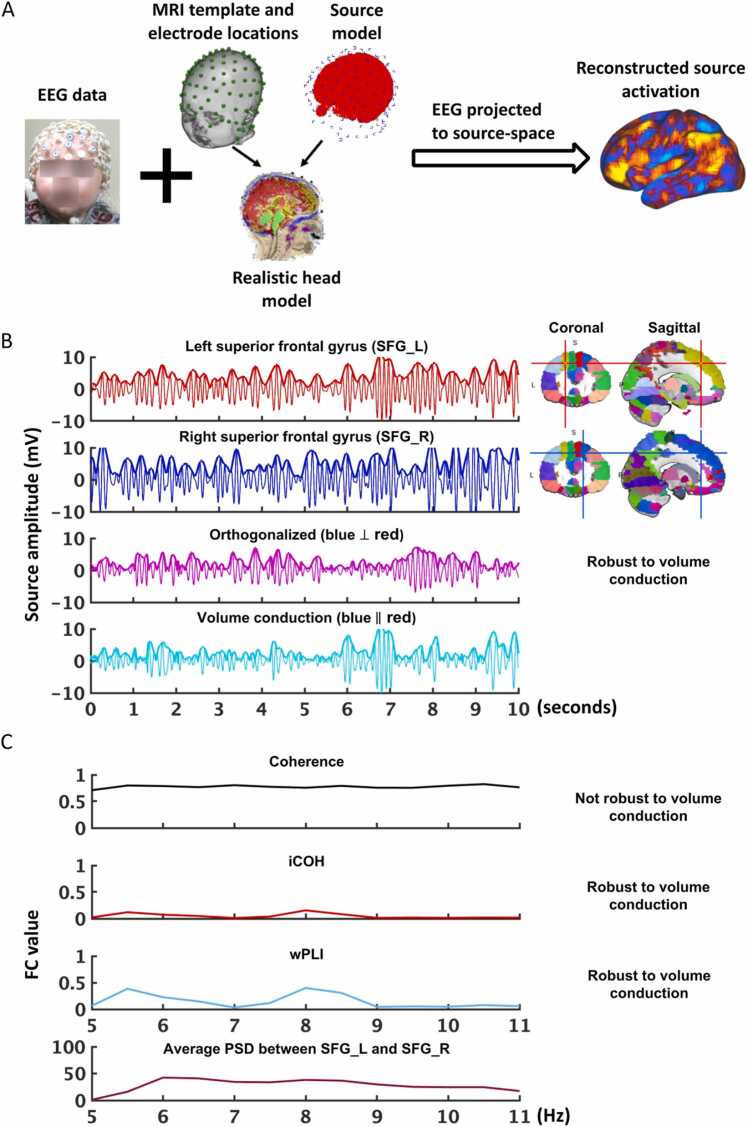
**Panel A** shows the process of cortical source reconstruction, which is conducted with the child EEG data and a realistic head model, and as a result, the scalp-level EEG data is projected into the source-space. **In panel B**, alpha band filtered (5–11 Hz) source-space time-series (10 s) in the left and right superior frontal gyrus (SFG), randomly selected from one participant’s data, are plotted in red and blue respectively. Applying the orthogonalization method to the two signals generates a new signal (magenta). In this figure, the signal in the left SFG that is orthogonalized to the time-series of the right SFG (blue) is calculated and plotted in magenta. This new signal is free of the zero-phase-lag effect. The signal in the left SFG that is likely caused by volume conduction (cyan) can be calculated by subtracting the orthogonalized signal (magenta) from the original signal (red), i.e., “cyan” = “red” – “magenta”. The correlation (corr1) between the natural logarithm of power envelopes of the “blue” and “magenta” signals is calculated, and the same kind of correlation (corr2) is calculated between the “red” signal and the signal in “blue” that is orthogonalized to “red”. The FC (AAC) in the alpha band between left and right SFG is the average of corr1 and corr2. **Panel C** depicts the FC (PPS) in the alpha band between the two regions using the source-space data from the same participant. Results from two robust methods, iCOH (red) and wPLI (cyan) and a not robust method, coherence (black) are plotted, along with the power spectrum density (PSD) (brown) in the alpha band. The two robust methods yield very similar patterns with two peaks in the alpha band for this participant, while using coherence yields generally higher FC values but in a different pattern with no clear peak in the alpha band.

In the current study we aimed to demonstrate two recently developed pipelines by two different research labs to conduct brain FC analysis in the source space, focusing on PPS (Xie et al., 2019a, Xie et al., 2019b) and AAC (Toll et al., 2020, Zhang et al., 2021) respectively (Fig. 1). For the sake of brevity, the two pipelines were referred to as the “pl_pps” and “pl_aac” in the following sections. The overall analytic streams of the pl_pps and pl_aac can be summarized into the following steps: 1) cortical source reconstruction with high-density EEG data and realistic MRI models; 2) parcellation of reconstructed source activities into brain ROIs for major brain lobes or brain networks; 3) calculation of FC between each pair of the brain ROIs or between source voxels/vertices in limited frequency bands using “robust FC metrics”, i.e., weighted phase lag index (wPLI; Vinck et al., 2011) and orthogonalized power envelope correlation (Hipp et al., 2012). Robust FC metrics refer to those measures that were designed to minimize the effects of spurious connections caused by volume conduction. The measure of wPLI was developed based on PLI, a method that discards the phase difference of zero or Pi by averaging the sign of the estimated phase difference, which is motivated by the fact that zero-phase difference is most likely to be caused by volume conduction (Stam et al., 2007). However, using PLI risks missing true instantaneous interactions and underestimates the connectivity at small-time lags and low signal-to-noise ratio (Cohen, 2015). In contrast, wPLI is less sensitive to noise and shows a more reliable relationship with true phase consistency because in wPLI the contribution of the observed phase difference is weighted by the magnitude of the lag rather than disregarding all zero-phase differences (Vinck et al., 2011). The orthogonalized power envelope correlation is another robust FC method that relies on correlations between oscillatory signals’ instantaneous amplitude across regions (termed power envelopes). To this end, orthogonalization between two time series (e.g., X and Y) needs to be performed, i.e., to compute the orthogonalized Y′ to X and vice versa, before the calculation of the correlation between their power envelopes (Fig. 2) (Hipp et al., 2012), with the assumption that orthogonalization yields a new signal (e.g., Y′) free of zero-phase-lag effect of the other signal (e.g., X).

While the main steps overlapped between the two pipelines, differences existed in their procedures of head model construction, frequency analysis, FC calculation and voxels parcellation, etc. Moreover, pl_pps primarily relies on functions in the Fieldtrip toolbox (Oostenveld et al., 2011), whereas pl_pps is mainly based on functions in the Brainstorm toolbox (Tadel et al., 2011). The examination of how these factors might influence FC outputs is out of the scope of the current study but has been conducted with adult EEG data (Mahjoory et al., 2017).

In this study we performed analysis following each pipeline with example data collected from 12- and 36-month-old children. Age-appropriate MRI templates were used to create the head models (O’Reilly et al., 2021, Richards and Xie, 2015). The two pipelines utilized functions in Fieldtrip (Oostenveld et al., 2011) and Brainstrom (Tadel et al., 2011), as well as customized MATLAB (R2020b, the Mathworks, Inc.) programs. Given there is a need in the research community for open-access processing pipelines and analytic toolkits that can be used for brain FC analysis with pediatric EEG data, programs and codes used for the current analyses have been made available to the public (see *Codes and Data Availability*).

## 2 Method

### 2.1 Participants

The sample data involved two different cohorts of children (N = 30 each at 12 and 36 months of age respectively. These children were randomly selected from an ongoing longitudinal study on the development of emotion processing in childhood. The demographic information (e.g., race/ethnicity; family combined income, and parental education) of the original cohorts has been described elsewhere (Xie et al., 2021). Parents of the participants provided written informed consent before their child’s study visits, and ethical permission for the study was obtained from the Institutional Review Board at Boston Children’s Hospital.

### 2.2 EEG data collection and preprocessing

EEG baseline (“resting-state”) data was recorded from a 124-channel HydroCel Geodesic Sensor Net (HGSN) connected to a NetAmps 300 amplifier (Electrical Geodesic Inc., Eugene, OR) while children watched a toy animation with sounds for 2 min. The EEG recording was referenced online to a single vertex electrode. Channel impedance was kept below 100 kΩ and signals were sampled at 500 Hz.

EEG recordings were preprocessed using EEGLAB (Delorme and Makeig, 2004) and customized MATLAB programs. The continuous EEG data were filtered with a Hamming windowed finite impulse response (FIR) filter with a passband of 1–50 Hz. The low-pass cut-off was set to be below the high gamma band to reduce the effect of the muscle- and movement-related artifacts on pediatric EEG data. The filtered data was then segmented into 2 s epochs. Independent component analysis (ICA) was conducted, and then SASICA (Chaumon et al., 2015) was used to identify and remove artificial components related to eye movements, blinks, and focal activity. The EEG epochs were then inspected for artifacts (EEG > 100 μV or EEG < − 100 μV). Channel interpolation was conducted using a spherical spline interpolation with the EEGLAB function “eeg_interp” if there were fewer than 18 (15 %) electrodes that were missing or had bad data. Each child must have at least 30 clean epochs (60 s) to be included for further analyses. The preprocessing procedures and the parameters used for each step were determined based on previous studies with pediatric EEG data (e.g., Xie et al., 2018, Xie et al., 2019a, Xie et al., 2019b). The scripts used for preprocessing are also shared with the FC analysis codes (see the ReadMe.md).

Given the dramatic changes in peak frequency of different rhythms in childhood (Marshall and Meltzoff, 2011, Perone et al., 2018), the preprocessed EEG data were filtered into four age-appropriate frequency ranges: theta (12mos: 3–6 Hz; 36 mos: 3–7 Hz), alpha (12mos: 5–10 Hz; 36mos: 6–11 Hz), beta (11–22 Hz for both ages) and gamma (22–45 Hz for both ages) bands. The boundaries of the low-frequency bands were made to be slightly overlapping because young children have large inter-individual variability in the frequency range of different bands, especially the theta and alpha bands. Since we were not using individual alpha peak to define the frequency boundaries in this tutorial, which should be the ideal method, we applied a wider range and slightly overlapped boundaries to better capture the theta and alpha activity for the participants. The filtering procedure was done before source localization to alleviate the influence of low-frequency oscillations on the source localization results (Xie et al., 2018, Zhang et al., 2021). In the pl_pps, the filtering process was conducted with the MATLAB FIR “bandpass” function with a steepness of 0.85, and then Hilbert transform was applied to the filtered EEG time series to obtain the complex-valued representation of signals, and thereafter the amplitude and phase time series were used in subsequent analyses. In the pl_aac, this was done by using the EEGLAB “pop_eegfiltnew” function with the default FIR parameters and a complex Morlet wavelet filter (similar to the Hilbert transformation).

### 2.3 EEG FC analysis in the source space

#### 2.3.1 Head model construction and source localization

Realistic head models were used in both pl_pps and pl_aac. In the pl_pps the head models were created for the 12- and 36-month-old cohorts separately using age-matched average MRI templates selected from the Neurodevelopmental MRI Database (Richards et al., 2016, Richards and Xie, 2015). Anatomical MRI templates were segmented into component materials, and a forward model was created for each age group using the Finite Element Method (FEM) with the gray matter being used as source volumes (6 mm grids), using the “ft_prepare_headmodel” and “ft_prepare_sourcemodel” functions in Fieldtrip with the following parameters: cfg.method = ‘simbio’, cfg.grid.resolution = 6, and cfg.grid.unit = ‘mm”. Age-appropriate skull conductivity values (0.066 Ω m^−1^ for the 12-month model and 0.036 Ω m^−1^ for the 36-month model) were used to build each forward model (Hämäläinen et al., 2011), using the cfg.conductivity option in the “ft_prepare_headmodel” function. The forward model was then used to estimate the lead-field matrix and the spatial filter matrix, i.e., the inverse of the lead-field matrix, using the “ft_prepare_leadfield” function in Fieldtrip. Distributed source reconstruction of the EEG time-series was conducted with the exact-LORETA algorithm (eLORETA; Pascual-Marqui et al., 2011, Pascual-Marqui et al., 2011) as the constraint for inverse modeling, using the “mkfilt_eloreta.m” function in Fieldtrip. In the pl_pps, we also provided an option for using the minimum norm estimation (MNE) algorithm for source reconstruction, by changing cfg.methodtype to be ‘MNE’. Singular value decomposition (SVD) was conducted to project the 3-dimensional time series in each voxel (i.e., dipole) along the direction (i.e., moment) that explains the most variance, using the “svd.m” function in Matlab. This projection is equivalent to determining the largest temporal eigenvector of the 3-dimensional time series.

In the pl_aac analyses, realistic head and brain surface models (O’Reilly et al., 2021) for each age group were generated via the Brainstorm neuroimaging toolbox for MATLAB (Tadel et al., 2011) incorporating appropriate conductivities for the various tissue types as described above. After down-sampling the cortical tessellation to 5000 vertices and fitting the appropriate EEG cap montage to the scalp surface, the lead-field matrix was calculated using the symmetric boundary element method (BEM) as employed in the OpenMEEG toolbox (Gramfort et al., 2010). The spatial filter matrix was obtained by weighted-MNE without noise modeling. To improve accuracy, this matrix was calculated without normal cortical restraint and reduced using principal components analysis of various frequency bands of the EEG data in each subject. Please see the ReadMe.md file and the “babyPublishOrthlog.m” program for details, as well as Fig. 2A for a summary of the source localization procedures.

#### 2.3.2 Parcellation of source voxels/vertices into ROIs

The complex-valued time-series in the source voxels or vertices were segmented into brain regions of interest (ROIs). This was done to ground our EEG source regions in commonly used, MRI (anatomical) derived definition of the major brain lobes or networks while maintaining ROI sizes large enough to be transformed for use in the lower-resolution electrophysiological context (Power et al., 2010).

In the pl_pps the 3D source volumes were segmented into 48 cortical ROIs using the LPBA40 brain atlas (Shattuck et al., 2008). The reconstructed time-series in the source volumes (voxels) surrounding the centroid of each ROI were averaged to represent the source activation for each ROI (Hillebrand et al., 2016; also see Hillebrand et al., 2012 for using the voxel with the greatest power). These ROIs were further grouped into the four major lobes, i.e., the frontal, temporal, parietal and occipital lobes. In this pipeline, we also provided other options for using the average activity or principal component analysis (PCA) to obtain the representative source activity for each ROI (Bathelt et al., 2013, Wirsich et al., 2021). These methods used to define the representative activity of an ROI have their own advantages and disadvantages. Using the component with the highest weight from PCA or average activity across voxels of an ROI may have a higher signal-to-noise ratio compared to using the centroid voxel or a few voxels surrounding the centroid; however, phase information can be distorted by the PCA or the average process, as the phase angles between oscillations of distant voxels are likely to be different. We recommend using one or only a few voxels surrounding the centroid to represent the activity of an ROI when PPS is the choice for FC analysis, although which method is the most suitable is still an open question for debate.

In the pl_aac, connectivity matrices of all pairwise connections were generated among all source vertexes and a t-test of each vertex for global correlation significantly greater than the mean across subjects was calculated. These values were used to identify connected vertices that emerged as hubs of significant connectivity. In this way functional ROIs were produced in a data-derived manner, agnostic of any atlas computed a priori. Mean connectivity from these ROIs, identified in each frequency band and in each age group, was then calculated to identify connectivity topologies (Toll et al., 2020). To the best of our knowledge, this is the first EEG-derived functional connectivity atlas produced and therefore yields interesting insights but would benefit greatly from validation and replication in larger datasets.

#### 2.3.3 Estimation of brain FC

In the pl_pps, the first step was to conduct frequency analysis with the Fieldtrip function “ft_freqanalysis”. In the current pipeline, a “hanning taper” was used, but it is also possible to use the multiple tapers based on discrete prolate spheroidal sequences (DPSS) by changing cfg.taper from “hanning” to “dpss”. The frequency analysis method was set up by defining cfg.method as “mtmftt”, which analyzed the entire spectrum for the entire data length. The output parameter “cfg.output” was set as ‘fourier” to generate the complex Fourier spectra. Please see step 4 “Frequency analysis of the source-space ROI data” in the “Intermediate outputs” section of the ReadMe.md file and the “SourceSpaceFCAnalysis_PPS” program for details.

The second step is to calculate the FC between ROIs in different frequency bands, using the Fieldtrip function “ft_connectivityanalysis” and the wPLI method (fcmethod = ‘wpli’; cfg.method = fcmethod). In this step, random permutation of trials was applied to avoid the influence of number of observations (trials) on wPLI estimation, as wPLI values for signals with lower synchronization can be overestimated when calculated across a low number of observations or epochs (Vinck et al., 2011). To this end, for each participant, 30 trials were randomly selected from all the trials to calculate the wPLI value. This procedure was repeated for 50 times and the average wPLI value was calculated as the final estimate for the participant. Please see step 5 “Functional connectivity analysis” in the “Intermediate outputs” section of the ReadMe.md file and the Matlab program for how this was achieved. The FC analysis resulted in 48 × 48 weighted adjacency matrices, with each element in the matrix representing the connectivity between a pair of ROIs. The Fisher’s r-to-z transformation was applied to the values in the matrices to improve the normality of their distribution. Since the FC analysis in pl_pps was conducted in Fieldtrip, other PPS methods can also be selected, such as the imaginary part of the coherency (iCoh) and phase-locking value. To do so, cfg.fcmethod needs to be changed to ‘imag’ or ‘plv’ before running the “SourceSpaceFCAnalysis_PPS” program. The Fieldtrip website has detailed tutorial on how to use their ft_connectivityanalysis function. Similar FC results have been obtained between using wPLI and iCoh (Xie et al., 2019a, Xie et al., 2019b).

For statistical comparisons, a sparsity threshold of.5 (top 50 %) was applied to the matrices to retain the strong and eliminate the noise connections, as well as to keep the same number of connections across matrices (Bathelt et al., 2013). This procedure was completed in by running the “GenerageTxtFilesForStats.m” program. Please see the “Other programs” section in the ReadMe.md file for how to use this program. This program also calculated the average FC between ROIs in the four major lobes, i.e., frontal, temporal, parietal and occipital lobes, and generated a text file with the results for all participants in an age group. The effect of using different thresholds (e.g., 0.1, 0.2, 0.3, and 0.5) to build the adjacency matrix has been tested in a recent study, which found similar results among using different thresholds (Xie et al., 2019a, Xie et al., 2019b).

In the pl_aac the FC was calculated using the orthogonalized power envelope correlation between each pair of the vertices. The time-series containing the phase and amplitude information of each vertex was first iteratively orthogonalized with respect to the rest of the vertices. Power envelopes were then calculated from each of the orthogonalized analytical time series, followed by a natural logarithm transformation to render them a normal distribution (Hipp et al., 2012, Toll et al., 2020). Pearson’s correlation analysis was conducted between the orthogonalized power envelopes for each pair of the vertices. For comparison, the pl_aac also calculated the correlation coefficient between the plain (non-orthogonalized) power envelopes for the vertex pairs (Toll et al., 2020). Toll et al. (2020) compared the brain connectivity patterns between using orthogonalized vs. non-orthogonalized power envelopes and found much stronger and dominated local connectivity patterns when using the non-orthogonalized power envelopes due to the volume conduction issue. The large adjacency matrix that contains the FC values between each vertex pair could be used to generate the average FC between each pair of the ROIs. The calculation of orthogonalized power correlation between all vertices is more time-consuming than doing the calculation between each pair of the ROIs after parcellation (e.g., 3000–5000 vertices vs. 30–50 ROIs). Thus, we provided an option for estimating brain FC using orthogonalized power correlation between brain ROIs in the pl_pps. Please see the “Intermediate outputs” section in the ReadMe.md document for examples of main intermediate outputs generated by the two pipelines. Please also Fig. 2B as an illustration of the main procedures in pl_aac and 2 C as an illustration of PPS results using different methods.

## 3 Results

In the following sections we present the results obtained from using the two pipelines with EEG data of 12- and 36-month-old children. The EEG data have been shared along with the scripts (see Codes and Data Availability). We provide instructions in the ReadMe.md file on how to make figures to display the FC results with open-access software.

### 3.1 Brain FC results from pl_pps

**Fig. 3:**
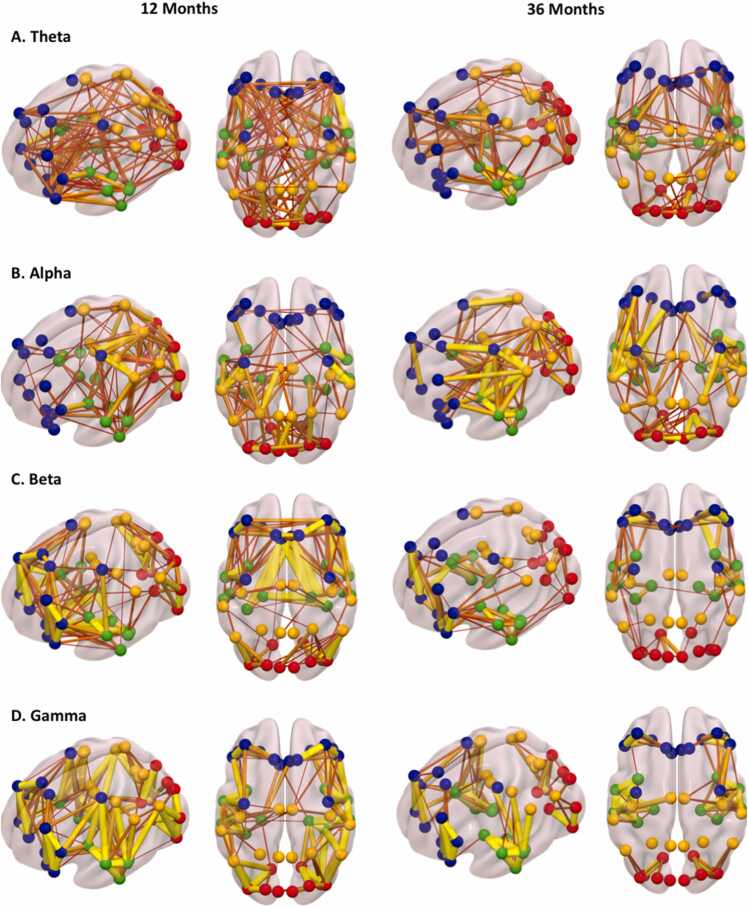
Functional connections between anatomical ROIs for the theta (A), alpha (B), beta (C), and gamma (D) bands using the pl_pps. The connections (edges) in this figure are transformed to z-values and plotted with the same threshold. Thicker lines are edges with stronger FC. Brain ROIs belonging to different lobes are plotted in different colors—frontal ROIs in blue, temporal ROIs in green, central and parietal ROIs in yellow, and occipital ROIs in red.

**Fig. 4:**
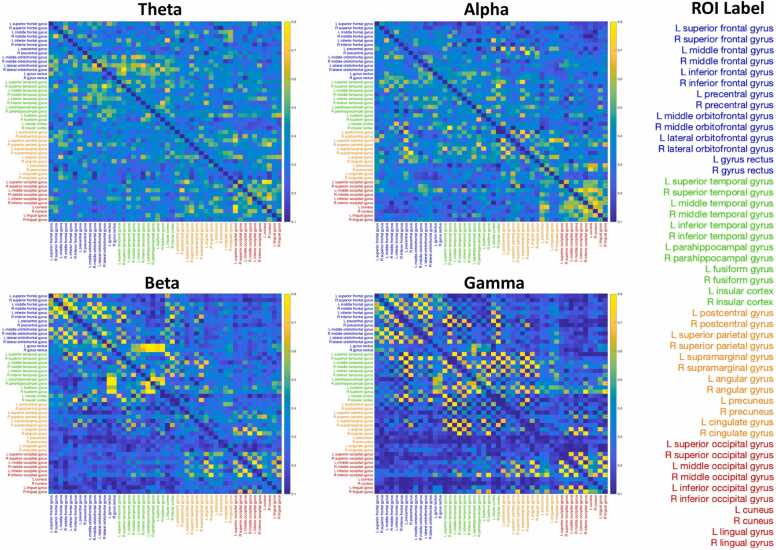
Adjacency matrices of functional connectivity in four different frequency bands, originated from the pl_pps using the wPLI method. The 48 cortical ROIs are divided into 4 lobes labeled in different colors: frontal regions in blue, temporal regions in green, central/parietal regions in orange, and occipital regions in red.

The FC analysis using the wPLI method in pl_pps resulted in four adjacency matrices (48 × 48 ROIs) for the four frequency bands. The ROIs were re-organized by which of the four major lobes the ROIs belong to, i.e., the frontal, temporal, parietal, and occipital lobes. We presented the corresponding averaged FC in a brain atlas using the Surf Ice software developed by Chris Rorden (Fig. 3). Brain FC showed different patterns across frequency bands and ages. Both long- and short-range connections were found for the theta bands at both ages. The hubs of FC in the alpha band were the central, parietal, and occipital regions. The FC in the beta band was most prominent among the frontal and temporal regions. A similar pattern of FC was found for the gamma band, except that strong gamma FC was also shown between the occipital regions. Another characteristic of the FC in the beta and gamma bands was that the brain modules seemed to comprise anatomically contiguous regions. Overall, brain FC decreased from 12 to 36 months, which possibly reflects the pruning of connections during childhood. The corresponding adjacency matrices for each brain FC figure can be found in Fig. 4. Quantitative analysis of the FC values can be achieved by parametric and nonparametric measures of the values in the adjacency matrices. We also provided a program to calculate the average FC between the main lobes (Tokariev et al., 2019, Tokariev et al., 2019) and save the outputs in a text file, using the program “GenerateTxtFilesForStats.m”.

**Fig. 5:**
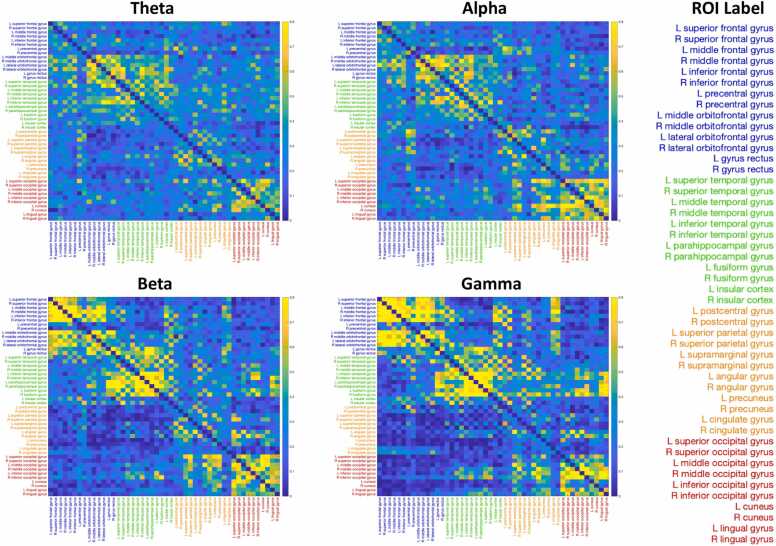
Adjacency matrices of functional connectivity in four different frequency bands, originated from pl_pps using the orthoganalized power envelop correlation method. Note: this figure was generated by the alternative option (i.e., SourceSpaceFCAnalysis_AAC.m) in pl_pps. It is not the output from pl_aac. The output from pl_aac is depicted in the following figure (i.e., Fig. 6).

There was an option for calculating the orthogonalized power envelope correlation using the pl_pps. This method resulted in more segregated network organization, i.e., the hubs were more localized, compared to the results from using wPLI. The adjacency matrices of orthogonalized power envelope correlation can be found in Fig. 5.

### 3.2 Brain FC results from pl_aac

**Fig. 6:**
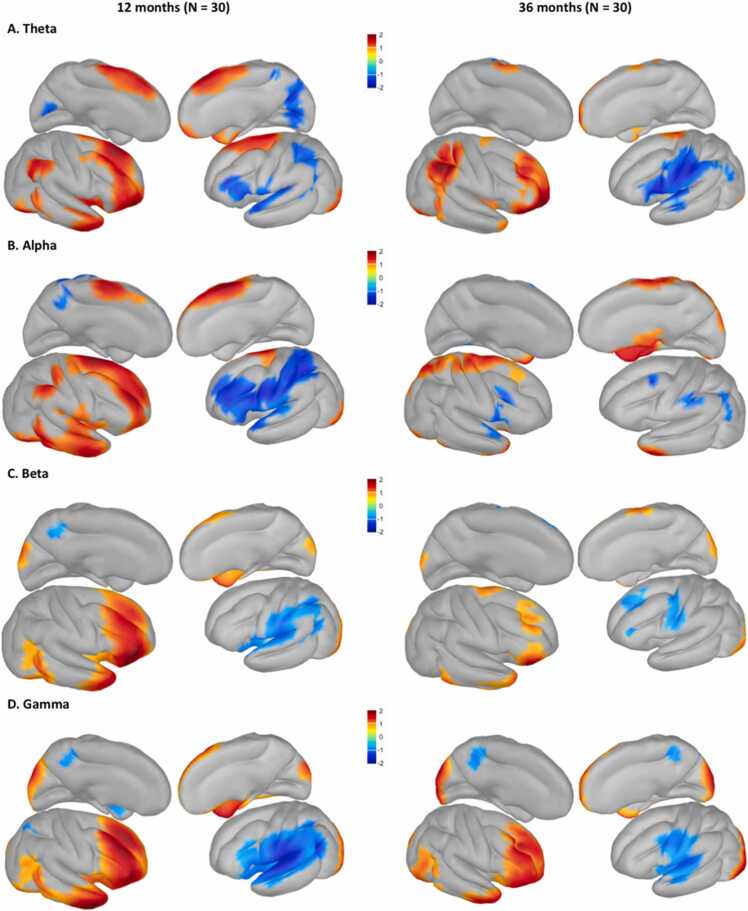
Functionally-derived connectivity hub regions for the theta (A), alpha (B), beta (C), and gamma (D) bands using the pl_aac. The red regions represent the clusters of vertices with relatively large magnitude t statistics, identifying the regions in the brain serving as significant hubs of connectivity in each frequency band. The blue regions represent the clusters of vertices with relatively small magnitude t statistics, identifying regions showing weaker connections with other regions.

Four frequency bands were investigated: theta, alpha, beta, and gamma. In each band, the 5000 × 5000 connectivity matrices, representing the correlation of each vertex of cortical source space orthogonalized to every other vertex, were robustly normalized using the median and median absolute deviation. Then each vertex’s global connectivity strength across all 30 subjects in each group was assessed by a Student’s T test, yielding a 5000 × 5000 matrix describing each vertex pair’s connectivity strength. Finally, the mean of this matrix was taken to yield a single 5000 × 1 column vector describing the topology of the observed mean connectivity (Fig. 6).

It is important to note this procedure is completely agnostic of any a priori model or atlas. Distinct topologies of connectivity emerged in each band and were highly congruent between the two cohorts despite being independently generated (Fig. 6). In the theta band, hubs of connectivity manifested in areas observed in adults including the principal nodes of the executive and salience networks (e.g., prefrontal lobe, areas toward the back of the parietal lob, and frontoinsular regions). These regions have also been identified as hubs of FC based on infant resting-state fMRI data, such that after 9 months of age, hubs started to emerge in higher cognition-related regions, including the lateral frontal area, insula, posterior superior temporal sulcus, and superior parietal lobules (Wen et al., 2019). In both cohorts, these patterns were strongly lateralized to the right hemisphere. The alpha band topology was the most disparate between the cohorts with the infants presenting higher temporal lobe connectivity. The beta band produced the clearest illustration of what is putatively the pruning and specialization of functional networks over a child’s cortical development. The least variance between the cohorts was observed in the gamma band as expected since local region intraconnectivity is likely to change the least over developmental pruning.

## 4 Discussion

In this study, we provided an overview of two recently developed pipelines by two research labs on EEG source space FC analysis. Step-by-step descriptions were provided, and the codes used in our analysis were made available to the public. The two pipelines (“pl_pps” and “pl_aac”) comprised the construction of realistic head models and cutting-edge FC methods that were used to alleviate the effects of volume conduction that could otherwise dominate the FC found on the scalp. Comparable results were found regarding the FC hubs in the theta, beta and gamma frequency bands between the two pipelines in terms of the location of FC hubs and the developmental change of the FC strength from 12 to 36 months of age. A major difference between the two pipelines was that the results from pl_pps did not show the lateralization (to the right hemisphere) that was shown in the pl_aac results. These pipelines did differ with respect to the methods used for head model creation (BEM vs. FEM; surface vs. volume models), cortical parcellation (vertices vs. ROIs), and FC estimation (PPS vs. AAC), which might lead to divergent results in source-space FC analysis.

The creation of realistic head models with age-appropriate MRI templates is a critical step in our source-space FC pipelines when used for pediatric EEG data. There are substantial neuroanatomical changes of neural tissues over childhood, and the structure of a child’s brain differs greatly from an adult’s brain (Phan et al., 2018, Richards and Xie, 2015). Consequently, using a head model created with an adult’s MRI for children’s EEG data will likely mislocalize the source of EEG currents (Reynolds and Richards, 2009), which in turn would influence the FC estimation in the source space. A significant advance in cortical source analysis for pediatric participants is to use realistic head models created with individual MRIs or an age-appropriate MRI template (Hämäläinen et al., 2011, Xie and Richards, 2017). Age-appropriate skull conductivity values were utilized in the pl_pps given the fact that skull conductivity value changes drastically in childhood, which is much higher for infants than adults (Odabaee et al., 2014). Although systematic estimation of skull conductivity for children at different ages has yet to be conducted, attempts have been made to use higher skull conductivity values for EEG source localization and source-space FC analysis for pediatric populations (Hämäläinen et al., 2011, Xie et al., 2019a, Xie et al., 2019b).

Another advantage of our pipelines is that both of them used robust FC metrics that were designed to minimize the spurious connections caused by volume conduction. In contrast, there are less-robust FC metrics, e.g., coherence and phase-locking value, that are considerably more sensitive to the effects of volume conduction (source mixing), and thus often reflect rather simple properties of the data, for instance, the strength of the sources and the corresponding mixing of the sources (e.g., the EEG power measured on the scalp) (Colclough et al., 2016). The wPLI metric (Vinck et al., 2011) used in the pl_pps is an adjusted version of the original PLI metric (Stam et al., 2007) that evaluates the distribution of phase differences (lags) across observations. The fundamental idea of PLI is to discard the phase difference of zero or π by averaging the sign of the estimated phase difference, which is motivated by the fact that zero-phase difference is most likely to be caused by volume conduction (like the idea of iCOH). However, using PLI risks missing true instantaneous interactions and underestimates the connectivity at small-time lags and low signal-to-noise ratio (Cohen, 2015). In contrast, wPLI is less sensitive to noise and shows a more reliable relationship with true phase consistency because, in wPLI, the contribution of the observed phase difference is weighted by the magnitude of the lag rather than disregarding all zero-phase differences. The orthogonalized power correlation used in the pl_aac also is, by design, insensitive to the trivial zero-phase-lag connectivity due to volume conduction (Hipp et al., 2012). The correlation is calculated between oscillatory signal’s instantaneous amplitudes across regions (i.e., power envelopes) after the orthogonalization of each pair of the signals (e.g., channels, brain voxels, or ROIs) that yields new signals free of zero-phase-lag effects (Toll et al., 2020). The characteristics of these methods have been extensively discussed elsewhere (Bastos and Schoffelen, 2016).

The impacts of using different head models, source localization algorithms, and FC methods on the output connectivity metrics have been reported in the literature (Barzegaran and Knyazeva, 2017; Quanying Liu et al., 2018; Mahjoory et al., 2017). For instance, a study presented a comprehensive assessment of the consistency of source localization and FC metrics across different anatomical templates (MNI152 vs. Colin27), head models (BEM, FEM, and spherical models), inverse algorithms (wMNE, eLORETA, and beamforming), and software implementations (Brainstorm, Fieldtrip, and their toolbox) (Mahjoory et al., 2017). The results for source localization and FC estimation were found to be relatively unaffected by the choice of head models, whereas the inverse algorithms and software packages induced a considerable variability. Specifically, significant differences were found between the beamforming and distributed (eLORETA/wMNE) inverse solutions and between the packages with different algorithms and brain atlases implemented (i.e., Brainstorm, Fieldtrip, and the authors’ customized toolbox). While the authors claimed no evidence for the superiority of a particular methodology (Mahjoory et al., 2017), another study found that the FC networks were more similar to those observed with resting-state fMRI when eLORETA was used as the inverse solution, compared to MNE and LCMV (Liu et al., 2018). It is worth noting that all these studies were based on adult EEG data. It is unclear how different parameters used in the current two pipelines, i.e., pl_pps and pl_aac, would affect the FC metrics obtained from pediatric EEG data. Thus, future research should investigate the potential impacts of these factors on source-space FC with pediatric EEG data.

The vertices-based results from the pl_aac shed light on the potential hubs of brain FC in different frequency bands in infants and young children. Anatomical and fMRI-derived functional atlases have been available for humans, including infants, for some time. However, frequency band specific connectomes in the source space, derived from high temporal resolution EEG, have only recently emerged as new mathematical approaches. One of these approaches, orthogonalized power envelope connectivity, was applied in this population to take advantage of the higher SNR afforded by young children’s thin skulls to yield the first frequency band specific FC atlases in human infants. These EEG-derived FC atlases (Fig. 6) provide insights into the development of functional brain networks in early childhood and would benefit greatly from validation and replication in larger (longitudinal) datasets. Whether brain networks identified with source-space EEG would map onto those derived from fMRI has yet to be understood, especially for young children. While there are reports that large-scale functional brain networks derived from resting-state EEG are “moderately comparable” to those obtained through fMRI in adults (Liu et al., 2017, Wirsich et al., 2021), a recent study using intracerebral EEG recordings showed that unlike with resting-state fMRI, brain modules identified based on phase synchronization predominately comprised anatomically contiguous regions (Williams et al., 2021). Moreover, the architecture of functional brain networks is still developing in childhood and can be drastically different than the brain networks in adults (Eggebrecht et al., 2017). Hence, future investigation of source-space FC with longitudinal EEG data will be crucial for us to better understand the development of functional brain networks over childhood.

### 4.1 Limitations and cautions

There are a few limitations to keep in mind when using the current pipelines for source-space FC analysis. First, the two pipelines might be more optimal for high- and mid-density EEG with more than 60 channels than low-density EEG that has inadequate surface sampling. A handful of studies have shown the importance of having a sufficient electrode density and full coverage of the head’s surface for cortical source localization (see Michel et al., 2004 for review). Although young children’s heads are much smaller than adults, and source-space FC has been conducted for infants with low-density EEG (Tokariev et al., 2019, Tokariev et al., 2019), how the results would differ between using low- vs. high-density EEG for source-space FC analysis still needs to be assessed. An alternative method for low-density EEG is to conduct sensor-space FC analysis with techniques that would reduce the effect of source mixings, such as partial coherence field (Pascual-Marqui et al., 2011, Pascual-Marqui et al., 2011) and surface Laplacian (Carvalhaes and De Barros, 2015).

Second, the “robust FC metrics” and source localization techniques employed in the current two pipelines will only alleviate but not rule out the detrimental effects of volume conduction, i.e., there still can be spurious false-positive connectivities in the vicinity of true connectivities due to “signal leakage” during source reconstruction (Palva et al., 2018). This is because the estimation of thousands of sources with the data in a hundred electrodes will always be under-determined, i.e., residual signal leakage from one location to other locations will always characterize the source data. As a result, there can be output connectivities as unwanted by-products of a true interacting pair of sources, and the location of the two sources can be misestimated. This type of spurious connection also refers to “ghost interaction” and can be resolved by a novel approach that bundles connections into hyperedges by their adjacency in signal mixing (Wang et al., 2018).

Third, accurate source estimates of infant EEG also depend on the modeling of fontanels and sutures, especially for infants in their first postnatal year of life when the fontanels are not fully closed (Lew et al., 2013). It is unclear how the fontanels and sutures will impact source estimates differently for infants at various ages (e.g., 3, 6 and 9 months), which is worthy of being investigated by future research. While the approaches introduced here should also be applicable to infants younger than 12 months of age, interpretation of the FC results needs caution when the fontanels and sutures are not taken into account in source modeling.

Finally, we had to limit our approaches to the variation of only a few processing parameters that we deemed reasonable and representative of pipelines that are recommended in practice. However, EEG-based brain connectivity analysis can be performed in numerous different ways, and it is still an open question for which method(s) would generate the FC metrics closest to the ground truth, especially when dealing with pediatric EEG data. It is therefore important to not consider one set of measures as the sole solution to all the issues raised above but try to select the techniques that best fit the data.

## Declaration of Interests

The authors, Wanze Xie, Russell Toll and Charles A. Nelson, declare no conflicts of interest.

## Data Availability

The codes and data described in the current study can be found at Github: https://github.com/happytudouni/SourceSpaceFCAnalysis_DCN. Please download the “SourceSpaceFC” folder for all the materials (e.g., codes, data and links). The materials for the two pipelines are saved in separate folders referred to as “pl_pps” and “pl_aac”. Please read the ReadMe.md document for instructions on how to use the programs.

## Glossary. This glossary provides supplemental information on the technical terms referred to in the manuscript, whereas it is not an exhaustive list of all terms employed, nor does it include all possible definitions for each term; definitions in addition to or different from those reported in this glossary may be found in other sources

Functional connectivity (FC): The temporal coincidence of spatially distant neural activities that likely reflects the dynamic interregional communications inside the brain and is usually estimated by statistical relationship between the measures of neural activity (e.g., EEG oscillations, fMRI BOLD signals).
Coherence: A measure of the FC in the frequency domain between two signals, loosely speaking, the similarly between the power spectrum of two signals.
Volume conduction: The transmission of electric fields from a cortical source (or dipole) through biological tissues towards measurement electrodes. Consequently, the electric fields generated by one dipole can be “visible” at multiple electrodes due to the complexity in tissue types and conductivity values and the smearing of the field by the skull and scalp.
(Cortical) source analysis: A method/technique that aims to identify the loci of neural activity that generates the EEG voltage and power distribution on the scalp
Head Model: A model that describes the media inside the head with their relative electrical conductivity; and loosely speaking, it is the link between the source volumes and the electrical potentials on the scalp in source analysis
Forward model: A computational model created with the head model, an electrode map for where the electrodes are, and a source model that describes where the sources (dipoles) are. The forward model delineates how the activation generated by the dipoles propagates to the scalp and is computed independent of the actual EEG data.
Boundary Element Model (BEM): A type of head model that segments the head into hierarchical compartments with homogeneous conductivity profiles within a compartment
Finite Element Model (FEM): Another type of head model that defines the conductivity of individual voxels inside the head based on the material of the voxel. For example, gray matter, white matter, and cerebrospinal fluid would each have its own conductivity value in a FEM model
Lead-field matrix: The mathematical representation (matrix) of the forward model, which can be used to calculate the scalp distribution with the current of a given source or any combinations of sources. The inverse of the lead-field matrix is the spatial filter matrix.
